# A Magnetic Levitation System for Range/Sensitivity-Tunable Measurement of Density

**DOI:** 10.3390/s23083955

**Published:** 2023-04-13

**Authors:** Junhui Yu, Donghai Li, Chengxian Zhu, Qiran Ouyang, Chunyang Miao, Haidong Yu

**Affiliations:** 1 Key Laboratory of Flexible Electronics (KLOFE), School of Flexible Electronics (Future Technologies) & Institute of Advanced Materials (IAM), Nanjing Tech University (NanjingTech), 30 South Puzhu Road, Nanjing 211816, China; 2 Xi’an Institute of Flexible Electronics, Northwestern Polytechnical University, 127 West Youyi Road, Xi’an 710072, China

**Keywords:** MagLev system, tunable, separation, density measurement, multi-magnets

## Abstract

Magnetic levitation (MagLev) is a promising density-based analytical technique with numerous applications. Several MagLev structures with different levels of sensitivity and range have been studied. However, these MagLev structures can seldom satisfy the different performance requirements simultaneously, such as high sensitivity, wide measurement range, and easy operation, which have prevented them from being widely used. In this work, a tunable MagLev system was developed. It is confirmed by numerical simulation and experiments that this system possesses a high resolution down to 10^−7^ g/cm^3^ or even higher compared to the existing systems. Meanwhile, the resolution and range of this tunable system can be adjusted to meet different requirements of measurement. More importantly, this system can be operated simply and conveniently. This bundle of characteristics demonstrates that the novel tunable MagLev system could be handily applied in various density-based analyses on demand, which would greatly expand the ability of MagLev technology.

## 1. Introduction

Density is a fundamental property of materials that is critical in various physical and chemical processes. Its significance in applications such as separation [1,2,3], chemical reactions [4,5,6], quality control [7,8], and blood tests [9] has made it an essential topic of interest in materials science and engineering [10,11,12,13,14,15]. Despite the several techniques proposed to determine material density, they suffer from significant drawbacks. Certain well-known density measurement methods, such as hydrometers, density-gradient columns, pycnometers, oscillating-tube densitometers, suspended microchannel resonators, and hydrostatic weighing balances, require large sample volumes and have limited precision, making them incompatible with certain sample types such as gels, pastes, and gums [16,17,18,19,20,21,22]. Additionally, the required equipment for some of these techniques is bulky, expensive, and challenging to use in certain environments. Therefore, the need for a portable, convenient, low-cost, and accurate density measurement method that can be used in resource-limited environments or field settings is still apparent.

One emerging technology that has attracted significant attention for its potential to address these challenges is magnetic levitation (MagLev) [7,9,23,24,25,26,27,28,29,30,31,32,33]. MagLev is a density-based analytical technique that uses magnetic fields to levitate objects, allowing their density to be determined from their position in the field. It has several advantages over traditional methods, including portability, low cost, simple operation, and precise measurements through visual readout [8,13,14,15,33,34,35,36]. MagLev has already found numerous applications in fields such as separation, self-assembly, forensics, quality control, and bioanalysis, and its simplicity makes it particularly suitable for use in resource-limited environments or as a point-of-care diagnostic tool [22,37,38,39,40,41,42,43,44].

For the original MagLev method, an upright cylindrical container filled with a paramagnetic medium (e.g., an aqueous solution of manganese chloride, MnCl_2_) is used as a density indicator after loading the test samples. Two indistinguishable NdFeB magnets are separately placed on the top and bottom of the container (the like poles facing each other) [27]. With this vertically aligned configuration, the sample’s density can be measured in a relatively wide range (0.8–3 g/cm^3^) with a density resolution in the order of 10^−2^ to 10^−4^ g/cm^3^ per 1 mm for a given concentration of the paramagnetic medium. Subsequently, it is found that by tilting the vertically aligned MagLev structure, the resolution gradually increases with the increase of the tilt angle [29]. At the same time, with the support of the container wall, the maximum measurable sample density of this tilted MagLev could reach 23 g/cm^3^. For the limit case, the MagLev system can be tilted to a horizontally aligned configuration, which has the highest resolution down to the order of 10^−6^ g/cm^3^ [29]. Nevertheless, the measurable range is only in the order of 10^−4^ to 10^−5^ g/cm^3^ since the supporting force from the container wall can no longer affect the measurement process. This tilted configuration greatly improves the measurement ability of MagLev but also brings great difficulty in operation. Besides this, the ring magnets were also used to construct the axial aligned MagLev structure, which is more convenient for adding samples and paramagnetic medium to the container [31,32]. However, the density resolution of this axial MagLev structure is only in the order of 10^−2^ to 10^−4^ g/cm^3^, and the range is about 0–4 g/cm^3^. Although different kinds of MagLev systems can be applied in different situations, a high-resolution MagLev that can be operated conveniently according to the diverse requirements is more expected in practical applications.

Here, we designed a tunable high-resolution MagLev system using multiple detachable magnets to meet the diverse requirements. This system enables the measurements to be adjustable by changing the number of magnets, whose resolution can be set from 10^−5^ to 10^−7^ g/cm^3^, and the range can be changed from 10^−3^ to 10^−5^ g/cm^3^. At the same time, this system also takes advantage of axial MagLev, which can be operated simply and conveniently. These properties endow the multi-magnet MagLev (M-MagLev) system with great potential to be applied in various density-based analyses on demand.

## 2. Materials and Methods

### 2.1. Design of M-MagLev System

**Figure 1:**
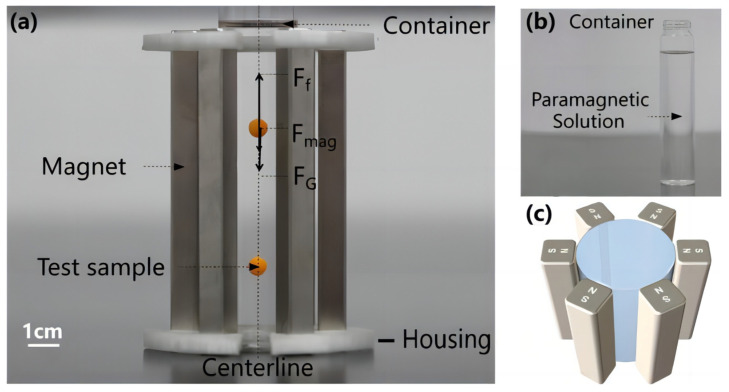
Overview of M-MagLev system. Photographs of (**a**) an M-MagLev system with 6 magnets and (**b**) the container in the M-MagLev system. (**c**) The corresponding simulation model of the M-MagLev system.

The M-MagLev system is designed based on the horizontally aligned MagLev structure to take advantage of high-resolution and straightforward operation. Here, an upright cylindrical container and several indistinguishable magnets make up the tunable system. As shown in Figure 1a,b, the paramagnetic medium and test samples can be conveniently added to the nonmagnetic container from the top opening. The detachable magnets surround the container symmetrically and are fixed by the magnet housing against the interaction between the magnets since the like-poles of the magnets face each other.

### 2.2. Theoretical Method

The samples levitated in a paramagnetic medium are subjected to the magnetic force *F_mag_*, the downward gravity force *F_G_*, and the upward buoyancy force *F_f_* in the vertical direction. The magnetic force, ${\vec{F}}_{mag}$, acting on the sample is given as:

(1) $${\vec{F}}_{mag}=\frac{\Delta \chi }{\mu_{0}}V\left(\vec{B}\cdot \nabla \right)\vec{B}$$

where V is the volume of the sample; $\mu_{0}$, the magnetic permeability of vacuum; $\vec{B}$ is the magnetic flux density; ∇ represents the gradient operator, i.e., $\nabla B\left(x,y,z\right)=\frac{\partial B}{\partial x}+\frac{\partial B}{\partial y}+\frac{\partial B}{\partial z}$; and Δχ is the magnetic susceptibility difference between the sample and paramagnetic medium. Here, we defined $\chi_{s}$ and $\chi_{m}$ as the magnetic susceptibility of the levitating sample and medium, respectively, so we have $\Delta \chi =\chi_{s}-\chi_{m}$. We set the centerline of the container as the *Z*-axis in the Cartesian coordinate system. As indicated by our previous work, the direction of the magnetic flux density at the centerline of the container is along the *Z*-axis, which is the best levitation position for the test samples [29]. According to the equilibrium conditions of force on the sample, we have:

(2) $$\left(F_{f}-F_{G}\right)+F_{mag}=\left(\rho_{s}-\rho_{m}\right)Vg+\frac{\Delta \chi }{\mu_{0}}VB_{z}B_{z}^{\prime }=0$$

where $F_{f}$ and $F_{G}$, respectively, are buoyancy force and gravity force on the sample; $\rho_{s}$ and $\rho_{m}$ the density of the sample and medium, respectively; g gravitational acceleration; $B_{z}^{\prime }$ the gradient of magnetic flux density B along the centerline. According to the equilibrium equation (Equation (2)), the density of the sample can be expressed as:

(3) $$\rho_{s}=\rho_{m}-\frac{\Delta \chi }{\mu_{0}g}B_{z}B_{z}^{\prime }$$

Under the effect of surrounding magnets, the magnetic flux density B along the centerline of the container will achieve two extremums: $B_{min}$ and $B_{max}$. Therefore, the maximum density $\rho_{smax}$ and the minimum density $\rho_{smin}$ that can be measured by the M-MagLev system could be determined as:

(4) $$\rho_{smax}={\rho_{m}-\frac{\Delta \chi }{\mu_{0}g}B_{z}B_{z}^{\prime }|}_{B_{z}=B_{max}}$$

(5) $$\rho_{smin}={\rho_{m}-\frac{\Delta \chi }{\mu_{0}g}B_{z}B_{z}^{\prime }|}_{B_{z}=B_{min}}$$

Here, the difference between the maximum density $\rho_{smax}$ and the minimum density $\rho_{smin}$ is defined as Δρ, which is the range of density measurements for the M-MagLev system with a certain number of magnets. After substituting $\rho_{smax}$ and $\rho_{smin}$, we have the expression of Δρ as:

(6) $$\Delta \rho =\rho_{smax}-\rho_{smin}\;=\frac{\Delta \chi }{\mu_{0}g}\left(B_{min}B_{min}^{\prime }-B_{max}B_{max}^{\prime }\right)$$

For a given concentration paramagnetic solution, the value of $\Delta \chi {\left(\mu_{0}g\right)}^{-1}$ remains constant. We introduce another important characteristic parameter, sensitivity (S), for the M-MagLev system to quantitatively indicate the resolution. The sensitivity is defined as the levitation height variation per unit density difference of sample with a unit of mm (g/cm^3^)^−1^. The larger S, the higher the resolution. So far, two key parameters to evaluate the performance of M-MagLev have been introduced here: measurement range Δρ and sensitivity S. To obtain the best performance, the numerical simulation for the tunable M-MagLev system is first conducted. Here, we use the COMSOL Multiphysics software to carry out numerical simulations and analyses based on theoretical equations.

### 2.3. Materials

We use the NdFeB bar-magnets (10 mm × 10 mm × 100 mm, strength N35) to structure the M-MagLev system in our validation experiments. They were purchased from Genchang Magnet Material Co., Ltd., Shanghai, China. We use the Gauss meter (HT108, Hengtong magnetoelectric technology Co., Ltd., Shanghai, China) to measure the magnets’ surface magnetic flux density (Bs). The magnet housings of M-MagLev system (the white polymer parts shown in Figure 1a) were fabricated by 3D printing (Shanghai Yinmeng Intelligent Technology Co., Ltd, Shanghai, China) with Nylon.

We used manganese chloride (MnCl_2_) aqueous solution prepared by MnCl_2_·4H_2_O and deionized water for paramagnetic medium. MnCl_2_·4H_2_O was purchased from Adamas-Beta Co., Shanghai, China. In some cases, NaCl_2_ could also be used to fine-tune the density of the solution. Therefore, the paramagnetic solution density $\rho_{m}$ could be adjusted according to the practical requirement, which determines the density benchmark of measuring objects.

Polystyrene beads were used as the test samples, which were purchased from Golden Ball Industry Co., Ltd., Ningbo, China. They were all about the same size (about 6 mm in diameter) and weight (about 0.11 g for each bead). We used a U-tube oscillating densitometer (DMA 35, Anton-Paar, Shanghai, China) to measure the density of the paramagnetic medium. Deionized water (18.4 MΩ/cm) used for all experiments was obtained from Milli-Q system (Millipore, Bedford, MA, USA). A digital single-lens reflex camera was used to record the levitation height.

## 3. Results

### 3.1. Simulation Results

**Figure 2:**
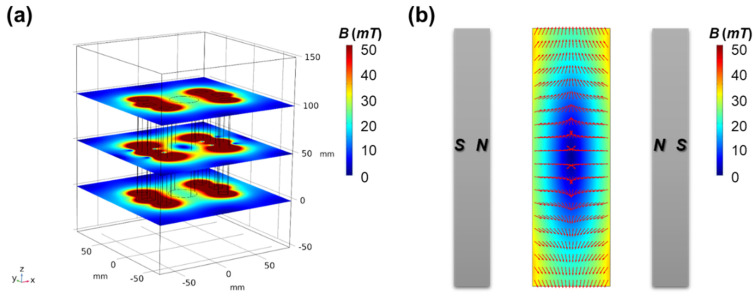
Simulation of the M-MagLev system with six magnets. (**a**) Contour plots of magnetic flux density distribution in horizontal slices of system. (**b**) Contour plots of magnetic flux density distribution in axis section of system. The red arrows indicate the direction of magnetic flux density.

The M-MagLev system with six NdFeB bar-magnets (10 mm × 10 mm × 100 mm) was chosen first for simulation. Six bar-magnets were arranged around the container, maintaining the same distance from the center of the container, with a radius (R, the distance between the magnets center and container center) of 42 mm. In order to simplify the analysis, we have imposed the restriction that the system exhibits only the simplest form of rotational symmetry, namely a C2 rotation symmetry, which implies that the system remains invariant under a rotation of 180 degrees around the center axis of the container. This assumption reduces the complexity of the problem and allows us to focus on the essential features of the system’s behavior. Furthermore, this symmetry allows the magnetic field distribution in the container to exhibit only one extremum point. Additional discussion on the distribution of the magnets can be found in Section 4. The magnetic flux density distribution of the M-MagLev system with six magnets is presented in Figure 2. To clearly illustrate the distribution of the magnetic flux density at different heights, we extracted three slices of the magnetic flux density distribution along the vertical direction, as shown in Figure 2a. It can be observed that the magnetic flux emanates from all magnets, and as it propagates, the magnetic flux density progressively declines. Inside the container, the magnetic fluxes mutually superimpose. At the central axis position of the container, due to the symmetrical distribution of the surrounding magnets, the in-plane magnetic flux components cancel out, forming a minimum point of magnetic flux density in the container region. Only the axial component along the container remains. Figure 2b shows the axial profile of the magnetic flux density norm in the container, where the red arrows indicate the direction of magnetic flux density. The distribution of magnetic flux density was found to be symmetric about the central axis, providing favorable magnetic field distribution conditions for accurately measuring the density of samples.

**Figure 3:**
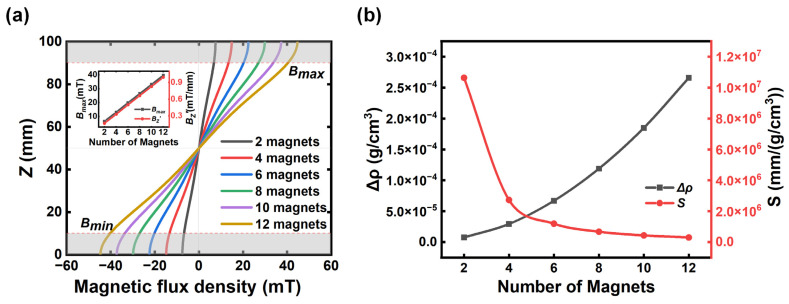
(**a**) Magnetic flux density profile along the centerline for the different numbers of magnets. The two gray areas at the top and bottom represent the parts blocked by the magnet housing. The inset presents the changes of *B_max_* and $B_{z}^{\prime }$ with the number of magnets. (**b**) Changes of range and sensitivity with the different numbers of magnets of the M-MagLev system.

According to the distribution of magnetic flux density, the central axis of the container is the most ideal measurement position. The distribution profiles of magnetic flux density along the centerline for a different number of magnets are summarized in Figure 3a. Except for the area hidden by the magnet housing (about 10 mm in thickness), the magnetic flux density along the *Z*-axis (*B_z_*) between the two housings changed almost linearly with the height of the container. Furthermore, *B_z_* was entirely antisymmetric about the middle point of the centerline where *B_z_* = 0 mT. Therefore, the area between the two housings was set as the density measurement area, and the distance between the two housings was defined as ∆Z. The maximum magnetic flux density (*B_max_*) locates at the top position of the measurement area. In contrast, the minimum magnetic flux density (*B_min_*) locates at the bottom position. They have the same size but opposite directions, i.e., $B_{max}=-B_{min}$. As we can see from Figure 3a, increasing the number of magnets from two to twelve, the gradient of magnetic flux density along the *Z*-axis ($B_{z}^{\prime }$) and two extrema increased gradually. The changes of $B_{z}^{\prime }$ and *B_max_* with the number of magnets are shown in the inset of Figure 3a. Both presented a linear change with the number of magnets. Accordingly, the measurement range Δρ and sensitivity S for the M-MagLev system can be simplified as:

(7) $$\Delta \rho =\frac{\Delta \chi }{\mu_{0}g}\left(2B_{max}B_{z}^{\prime }\right)$$

(8) $$S=\frac{\Delta Z}{\Delta \rho }$$

For the M-MagLev system with a different number of magnets, we calculated and summarized the range ∆*ρ* and sensitivity S with Equations (7) and (8) in Figure 3b. The increase in the number of magnets can greatly extend the measurement range of the M-MagLev, from $7.52\times 10^{-6}$ g/cm^3^ for two magnets to $2.66\times 10^{-4}$ g/cm^3^ for twelve magnets. In other words, the measurement range could be adjusted according to the practical needs just by changing the number of magnets in the M-MagLev system. On the other hand, the sensitivity decreases from $1.0\times 10^{7}$ mm/(g/cm^3^) to $3.0\times 10^{5}$ mm/(g/cm^3^) with the increasing number of magnets. By comparing with the typical horizontal high-resolution MagLev, this tunable system provides not only a higher resolution down to $3.3\times 10^{-6}\sim 1\times 10^{-7}$ (g/cm^3^)/mm, but a wider measurement range.

### 3.2. Experimental Results

**Figure 4:**
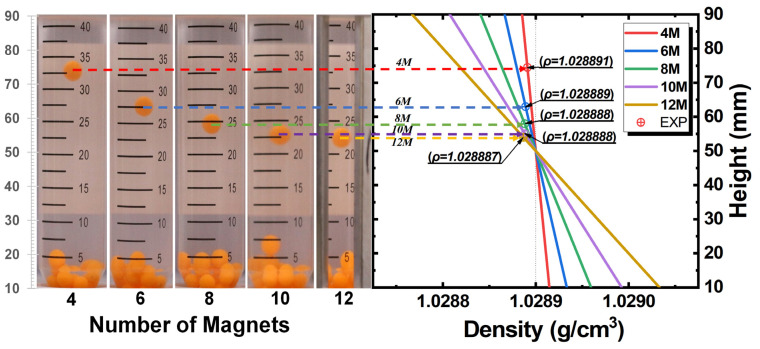
Density measurements using the M-MagLev system. Left part is captured photographs of the polystyrene bead levitated in M-MagLev with 4, 6, 8, and 10 magnets, respectively. Right part is the mapping of levitation height and the density of the bead for the corresponding M-MagLev according to the theoretical model. Symbols are the experimental results, while lines are the simulation results.

To verify the simulation results, we conducted proof-of-concept experiments of the M-MagLev system with a different number of magnets by levitating polystyrene beads in a container filled with MnCl_2_ aqueous solution (0.300 mol/L MnCl_2_·4H_2_O). The density of this paramagnetic medium $\rho_{m}$ can be calculated to be 1.0289 g/cm^3^, which is confirmed by the measurement of the U-tube oscillating densitometer. We selected several polystyrene beads for the test, whose densities were about 1.02 to 1.03 g/cm^3^, with slight differences in each other. If the bead density was beyond the range of the system, it would float on the top or settle down at the bottom of the container. For the levitated beads, the levitation height indicates the density of the bead: the higher position, the smaller density. As we can see in the left part of Figure 4, only one bead can levitate in the system. For M-MagLev with twelve magnets, this bead levitates near the center of the container. With the decrease in the number of surrounding magnets, the equilibrium position of this bead gradually rises. It indicates that the density of this bead is a little lower than 1.0289 g/cm^3^. The right part of Figure 4 presents the relationship between the levitation height and bead density with the number of magnets changing from 4 to 10. The lines are the simulation results, while the points are the experimental results. According to the levitation height, we can infer that the density of this bead is about 1.028891 g/cm^3^. Thus, the theoretical results are well-matched with the experimental results and provide reliable conclusions for the experimental results. Consequently, the tunable M-MagLev system is straightforward to operate and has a reasonably strong resolution to screen various samples, such as cells and drugs.

## 4. Discussion

### 4.1. The Linear Distribution of Magnetic Flux Density

**Figure 5:**
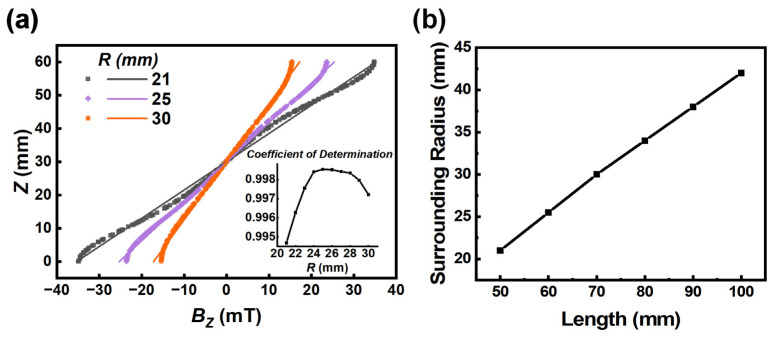
(**a**) Magnetic flux density profile along the centerline for different surrounding radii *R*. Points are the simulation results, and the corresponding lines were obtained by linear fitting. The coefficients of determination for the linear fitting are listed in the insert. (**b**) Relationship between the optimized surrounding radius and magnet length.

After systematic simulations, we found that the magnet’s length and the surrounding radius of magnets greatly influence the linear distribution of magnetic flux density along the centerline of the container (*B_z_*), which directly affects the accuracy and convenience of operation. Since the number of magnets has little effect on the distribution of *B_z_* (Figure 3a), we use the M-MagLev system with two magnets as an example to illustrate the effects. Firstly, we set the size of the magnet as 10 mm × 10 mm × 60 mm, where the length of the magnet is 60 mm. By numerical simulation, the change of distribution of *B_z_* with the surrounding radius R is plotted in Figure 5a. Naturally, a gradual decline in maximum magnetic flux density *B_zmax_* is presented in this figure as the magnets get farther and farther apart. However, the change of *B_z_* with height Z shows a certain degree of nonlinear distribution for some surrounding radii. Here, we use linear equations to fit these curves. The degree of linearity is evaluated by the coefficient of determination—the closer the value is to 1, the better the linearity of *B_z_*. As the insert in Figure 5a shows, with the increase of surrounding radius *R* from 21 mm to 30 mm, the coefficient of determination rises rapidly to the maximum value 0.9984 at *R* = 25 mm and then gradually decreases. That is to say, for the magnet 60 mm in length, a surrounding radius of 25 mm would give the distribution of *B_z_* the best linearity. Subsequently, we summarized the optimal radius for different magnet lengths in Figure 5b. A proportional relationship is presented between the optimized surrounding radius and magnet length. By fitting, it can be found that the linearity of *B_z_* was best when the ratio of surrounding radius to magnet length was about 0.4231. Accordingly, by choosing the appropriate surrounding diameter and length of magnets, better accuracy and convenience could be achieved for the M-MagLev system.

### 4.2. Measurement Stability

**Figure 6:**
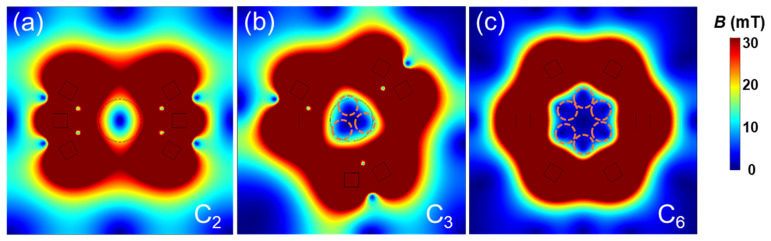
Magnetic flux density distribution in middle cross-section (x-y plane) of 6-magnet M-MagLev system with C_2_-symmetry (**a**), C_3_-symmetry (**b**), and C_6_-symmetry (**c**). The red dotted circles in (**b**,**c**) indicate the multiple stable positions.

To ensure that the direction of magnetic flux density on the container’s centerline is along the *Z*-axis, the magnets must be arranged around the container symmetrically. This part will discuss the influence of different symmetries on the M-MagLev system measurement results. Here, we still take the M-MagLev system with six magnets as an example. There are three kinds of axisymmetric distribution for the six magnets: C_2_, C_3_, and C_6_. Here, C_n_ is the rotational symmetry of order n, which means rotation by an angle of 360°/n does not change the object. By numerical simulations, we found that the symmetry of the magnet’s distribution does not affect the measurement range and resolution of the M-MagLev system but affects the levitation stability of samples. As shown in Figure 6, the distributions of the magnetic flux density at the middle cross-section (XY plane at the midpoint of centerline) for C_3_ and C_6_ symmetries present multiple stable points near the centerline (indicated by the red dot circles), which can easily lead to non-univocal results. For comparison, the distribution of magnetic flux density for C_2_ symmetry shows only one stable point. It lies on the container’s centerline, which is what operators expect for the practical measurement.

**Figure 7:**
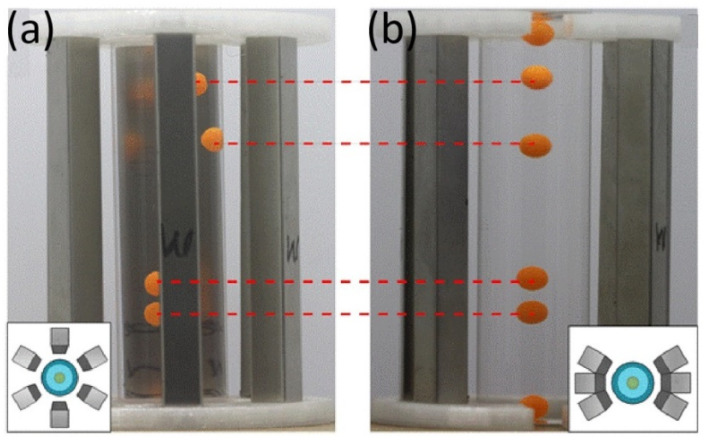
Captured photographs of polystyrene beads levitated in 6-magnet M-MagLev system with (**a**) C_6_-symmetry distribution of magnets and (**b**) C_2_-symmetry distribution of magnets. The insets are the distribution diagram of six magnets. The red dashed lines represent the different positions of the same ball under different magnetic distributions.

The corresponding experiments were also performed to verify the results. As indicated in Figure 7, the C_2_ symmetrical distribution of magnets makes all the levitated beads lie on the container’s centerline. In contrast, some beads are levitated off the centerline in the case of the C_6_ symmetrical distribution of magnets. This result is consistent with our numerical simulation and confirms that the C_2_ symmetrical distribution of magnets is beneficial to improve the measurement stability and accuracy of the M-MagLev system.

## 5. Conclusions

In summary, a novel tunable high-resolution MagLev system with multiple magnets was developed. By combining theoretical simulation and experiments, the mechanism and performance of this system were well demonstrated and studied. Compared with other MagLev structures, this M-MagLev possesses the highest resolution—down to 1 × 10^−7^ g/cm^3^. By changing the number of magnets, the resolution can also be changed from 10^−7^ to 10^−5^ g/cm^3^, and the corresponding measurement range varies from 10^−5^ to 10^−3^ g/cm^3^, which provides excellent convenience for connecting with other density measuring instruments with lower accuracy. With the systematic discussions on the basic parameters of the M-MagLev system, it is found that when the magnet length and surrounding radius reach a proportion of 0.4231, the magnetic flux density on the container’s centerline shows the best linearity with height. Meanwhile, the C_2_ symmetrical distribution of magnets can significantly improve the levitation stability of samples. These distinguished and adjustable advantages make this tunable high-resolution M-MagLev system suitable for a wide variety of density-based applications on demand.

## Data Availability

The data presented in this study are available on request from the corresponding author. The data are not publicly available due to internal ownership rules.
